# Exploring changes in children’s well-being due to COVID-19 restrictions: the Italian EpaS-ISS study

**DOI:** 10.1186/s13052-023-01521-9

**Published:** 2023-09-13

**Authors:** Marco Giustini, Ilaria Luzi, Angela Spinelli, Silvia Andreozzi, Mauro Bucciarelli, Marta Buoncristiano, Paola Nardone, Silvia Ciardullo, on behalf of the EPaS-ISS Group, Silvia Ciardullo, Paola Nardone, Marta Buoncristiano, Angela Spinelli, Marco Giustin, Silvia Andreozzi, Mauro Bucciarelli, Angela Giusti, Chiara Cattaneo, Ilaria Luzi, Amalia Egle Gentile, Francesca Zambri, Vittorio Palermo, Annachiara Di Nolfi, Gabriella Tambascia, Claudia Colleluori, Manuela Di Giacomo, Ercole Ranalli, Mariangela Mininni, Antonella Cernuzio, Francesco Lucia, Anna Domenica Mignuoli, Filomena Mortati, Gianfranco Mazzarella, Paola Angelini, Serena Broccoli, Marina Fridel, Paola Pani, Claudia Carletti, Federica Concina, Luca Ronfani, Lilia Biscaglia, Giulia Cairella, Maria Teresa Pancallo, Laura Pozzo, Camilla Sticchi, Federica Varlese, Corrado Celata, Olivia Leoni, Lucia Crottogini, Claudia Lobascio, Giusi Gelmi, Lucia Pirrone, Simona Chinelli, Giorgio Filipponi, Elsa Ravaglia, Stefano Colletta, Luca Belli, Martina Dichiara, Benedetta Rosetti, Marialuisa Lisi, Carla Patrizzietti, Stefania Matacchione, Ermanno Paolitto, Marcello Caputo, Pietro Pasquale, Giacomo Domenico Stingi, Pina Pacella, Maria Paola Ferro, Patrizia Miceli, Giacomo Lazzeri, Rita Simi, Carla Bietta, Marco Cristofori, Giada Fioretti, Federica Michieletto, Marta Orlando, Mauro Ramigni, Sabine Weiss, Pirous Fatehmoghadam, Chiara Mocellin, Maria Grazia Zuccali

**Affiliations:** 1 Environment and Health Department, Italian National Institute of Health, Rome, Italy; 2 National Centre for Disease Prevention and Health Promotion, Italian National Institute of Health, Rome, Italy; 3 Formerly National Centre for Disease Prevention and Health Promotion, Italian National Institute of Health, Rome, Italy; 4 “OKkio alla SALUTE” Technical Committee, Rome, Italy

**Keywords:** COVID-19, Well-being, Stay at home orders, Children, Primary school

## Abstract

**Background:**

While existing research has explored changes in health behaviours among adults and adolescents due to the COVID-19 outbreak, the impact of quarantine on young children’s well-being is still less clear. Moreover, most of the published studies were carried out on small and non-representative samples. The aim of the EpaS-ISS study was to describe the impact of the COVID-19 pandemic on the habits and behaviours of a representative sample of school children aged mainly 8–9 years and their families living in Italy, exploring the changes in children’s well-being during the COVID-19 pandemic compared to the immediately preceding time period.

**Methods:**

Data were collected using a web questionnaire. The target population was parents of children attending third-grade primary schools and living in Italy. A cluster sample design was adopted. A Well-Being Score (WBS) was calculated by summing the scores from 10 items concerning the children’s well-being. Associations between WBS and socio-demographic variables and other variables were analysed.

**Results:**

A total of 4863 families participated. The children’s WBS decreased during COVID-19 (median value from 31 to 25; *p* = 0.000). The most statistically significant variables related to a worsening children’s WBS were: time of school closure, female gender, living in a house with only a small and unliveable outdoor area, high parents’ educational level and worsening financial situation.

**Conclusions:**

According to parents ' perception, changes in daily routine during COVID-19 negatively affected children’s well-being. This study has identified some personal and contextual variables associated with the worsening of children’s WBS, which should be considered in case of similar events.

## Background

Coronavirus disease 2019 (COVID-19), the respiratory disease caused by the Severe acute respiratory syndrome coronavirus 2 (SARS‑CoV‑2) changed the daily routines of many families in Italy, severely affecting their economic stability and stress levels. Italy was the first European country to be hit by the first wave of the COVID-19 outbreak, declared a global pandemic by the World Health Organization (WHO) on March 11, 2020 [1]. Based on the risk assessment, on the same day, the Italian government imposed a total lockdown at the national level to control the spread of the virus and to reduce the burden on healthcare systems [2]. The adopted measures included closures of schools and educational institutions, sports facilities, gastronomy, and shops, except those selling crucial necessities, and cancellation of all sports events. Borders were partially closed, and travel was restricted. Employees and employers were strongly encouraged to switch to working from home where possible. The government-mandated physical distancing restrictions to reduce the spread of COVID-19 seem to have had a considerable impact on families’ health-related behaviours and lifestyles. Evidence regarding experiences from past outbreaks reveals that quarantine can create a substantial strain on the population and generate mental health problems [3, 4]. An epidemiological study reported that 17% of adults in the general population experienced Post Traumatic Stress Disorder (PTSD) symptoms during the initial stages of the pandemic [5]. While existing research has explored changes in health behaviours among adults and adolescents due to COVID-19 [5–8], the impact of quarantine on young children’s well-being is less clear. Nevertheless, emerging evidence suggests that home confinement, social restrictions, and prolonged school closure could have had severe consequences for children’s mental and physical health [9–13]. Moreover, a recent WHO report showed a worldwide modification in children’s behaviour and perceptions of their future, with 46% less motivated to do usual daily activities [14]. Some studies assessing well-being outcomes for children and adolescents have provided evidence of worsening outcomes in distance learning students compared to the pre-pandemic period or to students attending in-presence [15–18]. Other studies reported lower psychological well-being linked to COVID-19 in women [19], in people changing working conditions [20], with reduced income [18, 21, 22], and children with reduced levels of physical activity, longer screen time, irregular sleep patterns, and poorer eating habits [23].

In Italy, a web survey was carried out in the framework of the Project EPaS-ISS *Effects of COVID-19 pandemic on health behaviour and lifestyle of children and their families living in Italy*, promoted and funded by the Italian National Institute of Health (Istituto Superiore di Sanità, ISS), and coordinated by its National Centre for Disease Prevention and Health Promotion.

Within this project, the aim of the present study was to describe the impact of the COVID-19 pandemic and the consequent public health measures of physical distancing on some habits and behaviours of a representative sample of school-children attending the third grade of primary school (aged mainly 8–9 years) and their families living in Italy. Information about changes, perceived by parents, in the well-being of children between the pre-pandemic period and the pandemic period, was collected. General characteristics of children (gender and age) and parents (nationality, level of education, work organization, income) were investigated. Home characteristics (size and availability of outdoor areas) were also considered.

## Methods

Data collection, obtained from a web questionnaire, began in early April 2022 and finished at the end of September 2022. The target population was parents of children attending third-grade primary schools (8–9 years of age at the time the questionnaire was administered) and living in Italy. Data were collected according to a common protocol. Following the WHO European Childhood Obesity Surveillance Initiative (COSI) sampling approach, a two-stage stratified cluster sample design was used, with schools as primary sampling unites and classes as secondary sampling units [24, 25]. All parents of the selected classes were invited to participate. Sample extraction was performed from the primary schools previously involved in the 2019 “OKkio alla SALUTE” data collection, which is part of COSI. Seventeen Regions and two Autonomous Provinces of Italy were involved in the study. Regional and AP representatives, in collaboration with the Local Health Unit (LHU) ones, carried out and supported the activities at the local level. The EPaS-ISS study was presented to schools by Regional and LHU representatives with the support of Provincial School Offices. The parents of the enrolled classes received the information about the study as well as the link to access the web questionnaire from the headmasters and teachers.

The web questionnaire for parents was developed using LimeSurvey software and optimized according to the type of device used for its compilation (i.e., smartphone, tablet, personal computer). An information note with the description of the aim of the study and consent to the participation, a privacy policy for participation in the study and consent to the processing of personal data were also implemented online. The questionnaire, Information Note, and Privacy Policy were available online in Italian, English, Arabic and Chinese.

Parents were asked to report information about selected habits and behaviours. Among them, the following have been used in this study: parents’ and children’s age and gender, area of residence, nationality, educational level, family structure, financial situation, ways of carrying out work activities, size of the home in which the children spent most of the time during the COVID-19 period, number of children aged under 14 living in the home, availability of outdoor areas, months of school closures and interruption of sports activities. Parents were also asked to indicate any change between the *pre COVID-19 period* (before February/March 2020) and the *COVID-19 period* (from February/March 2020 to April 2022 based on the period of suspension of face-to-face school activities).

For both the pre COVID-19 and COVID-19 periods a Well-Being Score (WBS) has been calculated by summing the scores from the following 10 items rated on a five-point Likert scale (never, rarely, fairly often, very often, always): (1) Did your child feel well and fit? (2) Did your child feel full of energy? (3) Did your child feel sad? (4) Did your child feel lonely? (5) Did your child have enough time for himself/herself? (6) Was your child able to do the things he/she wanted to do in his/her free time? (7) Did your child feel that he/she was treated fairly by his/her parents? (8) Did your child have fun with his/her friends? (9) Did your child do well at school? (10) Was your child able to concentrate? The score of items 3 and 4 has been reversed. Internal consistency was assessed using Cronbach’s alpha [26]. The interpretation of alpha for a Likert scale question is as follows: (I) unacceptable if alpha < 0.5; (II) poor if 0.5 ≤ alpha < 0.6; (III) questionable if 0.6 ≤ alpha < 0.7; (IV) acceptable if 0.7 ≤ alpha < 0.8; (V) good if 0.8 ≤ alpha < 0.9; (VI) excellent if alpha ≥ 0.9.

Finally, the WBS has been rated again on a new five-point scale: 1 (Bad), 2 (Poor), 3 (Sufficient), 4 (Good), and 5 (Excellent). Cohen’s kappa has been used to assess the agreement in the WBS between pre COVID-19 and COVID-19. We assumed that the strength of agreement was (I) poor if kappa < 0.20; (II) fair if kappa = 0.21–0.40; (III) moderate if kappa = 0.41–0.60; (IV) good if kappa = 0.61–0.80; and (V) very good if kappa = 0.81-1.00 [27].

Socio-economic characteristics assessed in the study have been categorized as follows: respondents’ gender (male, female, other), children’s gender (male, female), area of residence (North, Centre, South of Italy), parents’ nationality (both Italians, at least one foreign parent), parents’ educational level (corresponding to the highest level between the two parents and classified as follows: low = both parents with less than high school, medium = at least one of the parents with high school, high = one of the parents with a university degree or higher), family structure (two-parent family, single-parent family), parents’ financial resources related to the COVID-19 period (worsened, unchanged, improved), parent’s ways of carrying out work activities (both on-site workers, at least one off-site worker). The characteristics of the home in which the children spent most of the time during the COVID-19 period were categorized as follows: dwelling size (less than 60 m^2^, 60–75 m^2^, 76–90 m^2^, 91–105 m^2^, 106–120 m^2^, 121–150 m^2^, more than 150 m^2^) and availability of outdoor areas (large habitable spaces, small non-habitable space, no outdoor space). The months of school closures and stops of sports activities were categorized as follows: never or less than one month (only for sports activities), 1–3 months, 4–6 months, 7–11 months, 12 months and more. Continuous data were presented as mean and standard deviation (SD) or median and interquartile range (IQR), as appropriate. All variables were summarized using frequency distributions. Differences in categorical variables between respective comparison groups were analysed using the χ^2^ test or Fisher’s exact test when expected cell counts fell below five. The Wilcoxon signed-rank test has been used for a repeated measure design where the same subjects are evaluated under two different conditions (pre COVID-19 period and COVID-19 period). Means or percentages and their 95% Confidence Intervals (95%CI) were calculated. Binary logistic regression was used to find the factors associated with the worsening in perceived well-being (worsened vs. unchanged or improved). The likelihood was described by Odds Ratios (OR) with their 95% confidence intervals (CI). Missing data and “Don’t know” responses were excluded from the analysis. The method used to calculate a CI for a proportion is the Clopper and Pearson exact binomial confidence intervals [28].

Weights to adjust for oversampling and nonresponse were used. All analyses took account of the clustered and stratified nature of the data. Analysis was performed using STATA (Stata Statistical Software: v. 15, Release 15, College Station, TX: Stata-Corp LP). A *p*-value of 0.05 or less was considered statistically significant.

## Results

Around 5900 parents gave their consent to participate in the survey, equal to 46.6% of families that were invited. Children with parents that answered just the few initial questions were excluded from the analysis. A total of 4,863 parents completed the questionnaire (mother: 89.1%; father: 10.5%; other: 0.4%) answering questions on 2,534 boys (52.1%) and 2,329 girls (47.9%), whose average age is 8 years and 9 months (SD ± 5 months). The parents’ characteristics are reported in Table 1. 

**Table 1:** Respondents’ characteristics

	Mother	Father	Other	*p* value
Area of residence				
* North*	47.4%	52.2%	52.6%	0.035
* Centre*	24.2%	25.9%	15.8%	
* South*	28.4%	21.9%	31.6%	
Nationality				
* Italian*	90.0%	91.5%	n.a.	0.000
* Foreign*	9.8%	7.3%	n.a.	
* Non-present person*	0.2%	n.a.		
Educational level				
* None*	0.4%	0.5%	n.a.	0.000
* Primary school (6 to 10 years)*	0.5%	1.2%	n.a.	
* Secondary school (from 11 to 13 years)*	12.0%	22.2%	n.a.	
* High school (from 14 to 18 years)*	46.9%	50.4%	n.a.	
* Degree*	31.8%	20.9%	n.a.	
* Master/Doctorate/Specialisation*	8.4%	4.8%	n.a.	
Employment				
* Housewife/homemaker*	24.6%	5.2%	30.7%	0.000
* Full-time worker*	41.0%	77.0%	23.1%	
* Part-time worker*	21.7%	7.3%	15.4%	
* Unemployed*	7.2%	3.5%	15.4%	
* Student*	0.0%			
* No employment due to illness or disability*	1.0%	0.5%	7.7%	
* Retired*	0.3%	1.1%	0.0%	
* Other*	3.2%	4.3%	7.7%	

The WBS was calculated for both pre COVID-19 and COVID-19 periods. Cronbach’s alpha exceeded 0.70 in both periods, ranging from 0.82 (pre-pandemic) to 0.85 (pandemic). The results indicate that the WBS has good internal consistency.

**Fig. 1:**
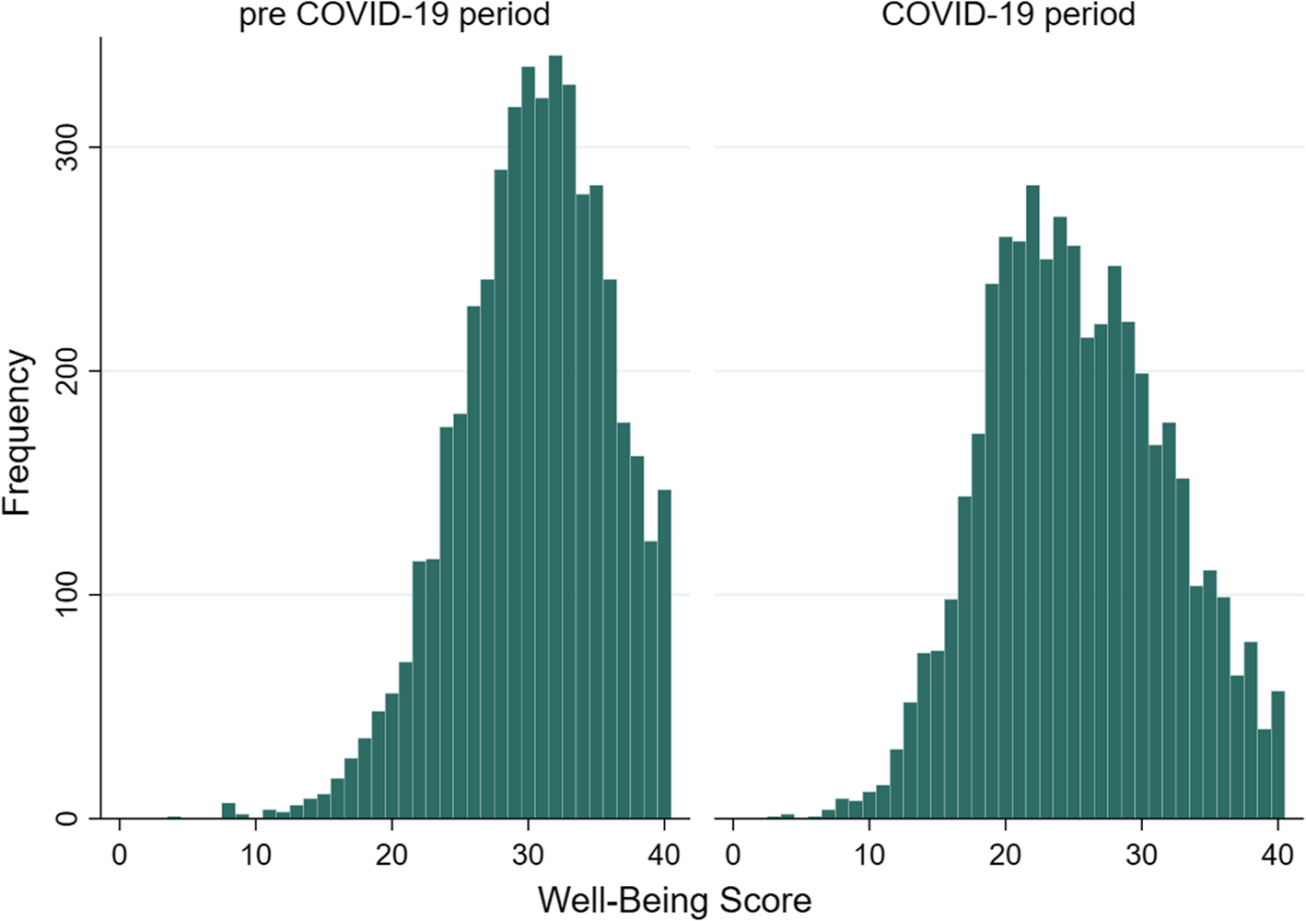
Distributions of the Well-Being Score (pre-COVID-19 period vs. COVID-19 period)

As expected, the WBS decreased from pre COVID-19 period (median value = 31, IQR = 7) to the COVID-19, (median value 25, IQR 10). According to the Wilcoxon sign rank test, the distributions of WBS scores per period show a statistically significant difference (*p* = 0.000). As shown in Fig. 1, both pre COVID-19 and COVID-19 period WBS distributions are leptokurtic (kurtosis: 3.29 vs. 2.54 respectively), but the former is negatively skewed (skewness: -0.52 vs. 0.09), i.e., shifted to higher values and with longer tail on the left side of the distribution.

Table 2 shows the shifting of the WBS pre and during the COVID-19 period as ranked in five classes. Only 3.7% of respondents reported an overall improvement in their child’s well-being. As expected, most (52.6%) reported a deterioration in their child’s well-being. Therefore, there is no agreement between the pre COVID-19 and the COVID-19 periods WBS ranked classifications (agreement = 43.7%; expected agreement = 28.7%; kappa = 0.2096; *p* = 0.0000).

**Table 2:** Well-Being Score agreement (pre COVID-19 period vs. COVID-19 period)

		COVID-19 period				
**pre COVID-19 period**		Bad	Poor	Sufficient	Good	Excellent
	Bad	0.11%	0.02%	0.00%	0.02%	0.00%
	Poor	0.04%	0.63%	0.33%	0.00%	0.00%
	Sufficient	0.02%	2.24%	9.95%	1.06%	0.17%
	Good	0.11%	3.34%	22.52%	20.11%	2.13%
	Excellent	0.09%	1.50%	7.38%	15.33%	12.88%

Table 3 shows the association between WBS shifting (worsened, unchanged, and improved) and domains linked to well-being. Except for dwelling size, all the domains are associated with the worsening of WBS. Fathers, as questionnaire respondents, have a less negative perception of change than mothers do (*p* = 0.000); the same is for families with at least one foreign parent (*p* = 0.000) and single-parent families (*p* = 0.007). Parents’ educational level is also associated with worsening perceived well-being, where parents with lower educational level show a higher perception of unchanged situation of WBS of their children seem to perceive their children as more resilient (*p* = 0.000). The data show a trend between months of school closure as well as months of stopping sports activities and the percentage of perceived worsening of children’s well-being: a longer period when schools are closed, or sports activities are discontinued, corresponds with a higher percentage of parents who perceive their child’s well-being as having deteriorated (*p* = 0.000). Parents whose financial situation improved are less likely to report a worsening in children’s WBS. Parents’ work organization is not statistically associated with a different perception of well-being (*p* = 0.517).

**Table 3:** Shifting of Well-Being Score due to COVID-19 by variables of interest

	Worsened	Unchanged	Improved	*p* value
Responder to the questionnaire				
* mother*	54.2%	42.2%	3.6%	0.000
* father*	39.0%	56.6%	4.4%	
* other*	47.4%	42.1%	10.5%	
Gender of the child				
* male*	50.5%	46.0%	3.5%	0.005
* female*	54.8%	41.2%	4.0%	
Parents’ nationality				
* both Italians*	55.2%	41.7%	3.1%	0.000
* at least one foreign parent*	37.6%	53.9%	8.5%	
Family structure				
* two parent family*	54.1%	42.4%	3.5%	0.007
* single-parent family*	47.8%	47.5%	4.7%	
Highest educational level among parents				
* low*	35.7%	57.1%	7.2%	0.000
* medium*	52.9%	43.0%	4.1%	
* high*	56.7%	40.5%	2.8%	
Geographic area of the school				
* North*	52.1%	44.6%	3.3%	0.006
* Centre*	56.2%	40.8%	3.0%	
* South*	50.5%	44.5%	5.0%	
Dwelling size				
* less than 60 square metres*	45.9%	47.2%	6.9%	0.189
* 60–75 square metres*	54.1%	42.2%	3.7%	
* 76–90 square metres*	51.9%	44.3%	3.8%	
* 91–105 square metres*	54.4%	42.9%	2.7%	
* 106–120 square metres*	54.1%	43.1%	3.2%	
* 121–150 square metres*	55.0%	42.5%	2.5%	
* more than 150 square metres*	52.7%	43.0%	4.3%	
Availability of outdoor areas				
* yes, large living spaces*	50.5%	45.6%	3.9%	0.000
* yes, small, unliveable spaces*	62.5%	35.2%	2.3%	
* no living space*	48.9%	44.8%	6.3%	
Children under 14 in the home				
* one*	51.0%	44.6%	4.4%	0.000
* two*	56.0%	40.8%	3.2%	
* three*	51.6%	43.9%	4.5%	
* four and more*	29.4%	66.4%	4.2%	
Months of school closure				
* less than three months*	31.8%	62.0%	6.2%	0.000
* four to six months*	51.6%	44.4%	3.9%	
* seven to eleven months*	58.0%	39.1%	2.9%	
* twelve months and more*	56.3%	39.7%	4.0%	
Months of stopping sporting activities				
* never, or less than one months*	35.6%	62.7%	1.7%	0.000
* one to three months*	46.8%	49.5%	3.7%	
* four to six months*	56.1%	41.6%	2.3%	
* seven to eleven months*	59.6%	38.3%	2.1%	
* twelve months and more*	64.1%	33.2%	2.7%	
Parents’ financial situation				
* worsened*	57.1%	41.9%	1.9%	0.000
* unchanged*	54.2%	43.2%	2.6%	
* improved*	62.2%	34.5%	3.3%	
Parents’ work organization				
* Both on-site workers*	55.5%	41.9%	2.6%	0.517
* at least one off-site-worker*	58.0%	39.4%	2.6%	

Table 4 reports the results of the logistic regression. The multivariate analysis includes significant variables for WBS shifting (see Table 3), namely responder to the questionnaire (i.e. kinship to the child), gender of the child, parents’ nationality, family structure, highest educational level among parents, geographic area of the school, availability of outdoor areas, number of children under 14 living in the home, months of school closure, months of stopping sporting activities, and parents’ financial availability. Due to the small number of children who improved their well-being (3.7%), these were considered together with children whose level of well-being remained unchanged. This allows for a binary outcome of comparable size (worsened vs. not worsened) while avoiding problems of low statistical power. The model shows the following predictors for worsening well-being when the other variables are kept constant: if the respondent is a mother (OR = 1.83, 95%CI = 1.39–2.40, *p* = 0.000), being a girl (OR = 1.23, 95%CI = 1.03–1.46, *p* = 0.019), having had at least four months of school closure (OR increases from 1.55 for 4–6 months of closure to 2.07 for 12 or more months of closure), living in the Centre of Italy (OR = 1.31, 95%CI = 1.06–1.62, *p* = 0.011), have at least one parent with medium educational level (OR = 1.62, 95%CI = 1.01–2.60, p = 0.047), have small, unliveable outdoor spaces (OR = 1.52, 95%CI = 1.03–2.24, *p* = 0.035) and have a worsened financial availability due to COVID-19 pandemic (OR = 1.41, 95%CI = 1.16–1.70, *p* = 0.001). Two other predictors of worsened WBS were found to be remarkably close to the statistical significance: stop of children’s sports activities for at least twelve months (OR = 1.97, 95%CI = 0.99–3.92, *p* = 0.053) and having at least one parent with high educational level (OR = 1.59, 95%CI = 0.99–2.55, *p* = 0.055).

**Table 4:** Model of binary logistic regression of worsening child’s WBS

Variables	Baseline level	OR	95% CI	*p* value
*Responder to the questionnaire: mother*	*father*	1.83	1.39–2.40	0.000
*Child’s gender: female*	*male*	1.23	1.03–1.46	0.019
*school closure (4–6 months)*	*1–3 months*	1.55	1.03–2.32	0.034
*school closure (7–11 months)*		1.92	1.28–2.88	0.002
*school closure (≥ 12 months)*		2.07	1.19–3.59	0.010
*stop sport (1–3 months)*	*no stop*	1.23	0.60–2.52	0.570
*stop sport (4–6 months)*		1.40	0.70–2.78	0.337
*stop sport (7–11 months)*		1.62	0.81–3.24	0.176
*stop sport (≥ 12 months)*		1.97	0.99–3.92	0.053
*single-parent family*	*two-parent family*	0.87	0.68–1.13	0.300
*at least one foreign parent*	*both Italians*	0.82	0.58–1.16	0.266
*geographic area of the school (Centre)*	*North*	1.31	1.06–1.62	0.011
*geographic area of the school (South)*		1.13	0.90–1.42	0.289
*educational level (medium)*	*low*	1.62	1.01–2.60	0.047
*educational level (high)*		1.59	0.99–2.55	0.055
*outdoor areas (large living spaces)*	*none*	0.92	0.64–1.32	0.653
*outdoor areas (small, unliveable)*		1.52	1.03–2.24	0.035
*children < 14 at home (two)*	*one*	1.15	0.95–1.40	0.154
*children < 14 at home (three)*		1.20	0.88–1.64	0.239
*children < 14 at home (four or more)*		0.55	0.28–1.11	0.094
*parents’ financial situation (improved)*	*unchanged*	1.26	0.83–1.90	0.283
*parents’ financial situation (worsened)*		1.41	1.16–1.70	0.001

## Discussion

This survey explored parents’ opinions about the children’s well-being during the COVID-19 pandemic. To the best of our knowledge, this study is one of the few to investigate the well-being of young children in a large sample [15, 29–32]. Consistent with recent research findings [20, 33], our data show parents’ negative perceptions of their children’s well-being associated with the COVID-19 pandemic. The results indicate that being a female (of both the respondent and the child), living in central Italy, having a medium-high educational level, the deterioration in financial situation and the closure of schools are predictors of children’s reduced well-being, according to the parents’ perceptions.

Consistent with other studies [34–37], our results suggest that females have been at higher risk of depression and anxiety symptoms and obsessive-compulsive disorders. This may explain the strong association of perceived worsening (OR = 1.83) with the female gender of the respondent.

Overwhelming scientific literature showed that school closure was one of the most disruptive circumstances for children during the COVID-19 outbreak [12, 38–43]. Indeed, children usually spend a large amount of their lives at school, which represents a central setting for their development; the school-based regular relationships with peers and teachers also play a key role in children’s well-being [44]. According to many authors [12, 45, 46], enforced social distancing was likely to result in increased loneliness that has been related to a greater chance of developing mental health problems. Consistent with the study of Verlenden et al. [16] and Pieh et al. [17], our results highlight a strong association between school closures and worsening well-being. In general, the severity of restrictions could also have consequences on the well-being of young children. Conversely, a study demonstrated that children in Sweden experienced low levels of anxiety largely because schools remained open [47]. On the one hand, keeping life as normal as possible could be one crucial factor in preventing the worsening of well-being in children during a pandemic. On the other hand, it would facilitate transmission and consequently more cases and deaths. Restrictions on sports activities and the requirement to stay at home also led to a reduction in outdoor physical activity and increase in sedentary behaviours [48]. Although not statistically significant in the logistic regression model, our results suggest that a prolonged interruption of outdoor sports activity may lead to a worsening of well-being.

Parental socioeconomic status has also importance for the health outcomes of children during the COVID-19 pandemic. A decrease of the financial resources of parents affected the families’ lives. Due to the serious economic recession caused by the COVID-19 pandemic, low-income families faced additional threats that may have negatively influenced children’s well-being [49–51]. Our study is consistent with these findings.

The relationship between dwelling size and well-being is commonly likely to be positive and we expected that confinement caused by the COVID-19 pandemic has made this association more important than in usual living conditions. According to Amerio et al. [52], poor housing is associated with an increased risk of depressive symptoms during lockdown. Living in apartments < 60 m^2^ with poor views and scarce indoor quality is associated with, respectively, 1.31 (95% CI: 1.05–1.64), 1.37 (95% CI: 1.17–1.61), and 2.25 (95% CI: 1.92–2.65) times the risk of moderate-severe and severe depressive symptoms. However, we did not find any association between the size of the living space and perceived well-being. According to Foye [53], adaptation theories suggest that space is a less important metric of societal well-being, having only a temporary effect on well-being and as individuals adapt, their well-being reverts to its previous level. Our study design considered “COVID-19” a time span lasting more than 24 months when people’s well-being perceptions could have time to adapt to the new status. Indeed, in Italy, the hardest lockdown occurred during the first wave (March-May 2020), whilst from June 2020 to April 2022 more flexible periods followed new periods of general closures and social distancing. It may be speculated that if the questionnaire had been administered during the first wave when people were experiencing the harshness of the lockdown, the size of the house would have played a key role in the perception of well-being.

More surprising in our study, the availability of a small, non-habitable outdoor area, rather than its absence, acted as a factor of worsened well-being (OR = 1.52). We would be led to believe that having an outdoor space, albeit small and not liveable, is better than not having it at all. It may be that in a time of restricted personal freedoms, having only a small and unliveable outdoor space has increased the regrets for not having a large and habitable one. Indeed, some authors [54, 55] found that the size of living space is likely to be a positional good: i.e., having a 90 m^2^ house in a world where everyone else has a 75 m^2^ house, is perceived as better than having a 120 m^2^ house in a world where everyone else has a 150 m^2^ house. However, it is possible that the WBS scale - made up of 10 questions that investigate many dimensions of well-being - is not overly sensitive to some determinants connected to the housing.

Consistent with other studies’ findings, our data support that the prevalence of lower child’s well-being is significantly influenced by the high parent’s educational level, perhaps due to the elevated self-awareness of their health [19, 56, 57]. We found a greater chance of worsening in perceived well-being in families of medium and high educational levels (OR = 1.62 and 1.59, respectively) compared to low educational level families.

## Conclusions

The COVID-19 pandemic has constituted a challenging experience not only for public health and economic stability but also for young children’s and parents’ well-being. Unexpected changes in daily routine negatively affect families’ living in Italy jeopardising the well-being of children. The EPaS-ISS study identifies some personal and contextual variables involved in the psychological adjustment to the social distancing measures that can help the healthcare system in the early detection of worsening in the level of well-being.

It will be interesting to monitor if these changes in perceived well-being of children remain and may have an effect on childhood lifestyle. In this regard, longitudinal studies focusing on parents’ and children’s well-being across many time points are needed to assess the long-lasting effects of the lockdown measures. Moreover, further research should review the polices and strategies adopted by other countries to promote positive change in lifestyles of children and their families. In terms of risk management policies, particular attention should be paid to the consequences of school closure and restrictions on sports activities, which seem to be among the main causes of the deterioration of the child’s well-being.

To the best of our knowledge, the EPaS-ISS is one of the few studies using a large, nationally representative sample to assess the level of perceived well-being among the target population of young children. The use of a large national sample from almost all Italian regions is definitely one of the firm’s strengths as well as the use of standardized data collection procedures based on the international COSI, and the support of Regional Coordinators and Local Health Unit personnel involved in the “OKkio alla SALUTE” surveillance system.

However, the current study had also some limitations: (1) being an Internet user, i.e. having basic technical skills such as being able to use computers, tablets, or smartphones, was required to take part in the investigation and could have been a source of selection bias: it is possible that disadvantaged families were less likely to participate in this study, and therefore, this may have led to lower representation from these groups and, in general, to low response rate [58]; (2) the evidence relied on parent reports because young children (8–9 years old) are still limited in their abilities to accurately describe a general well-being status. In our study, the children’s well-being assessment is the result of their parent’s opinion that may not match the true well-being of the child; (3) online surveys have several advantages, such as the opportunity to quickly reach a considerable number of participants, especially when it was important to maintain a social distance. However, the use of self-report measures in the online survey did not allow controlling for the accuracy of filling in the whole questionnaire and some questions may have not been well understood; (4) lastly, a score on a not still validated scale (WBS) was used to measure the change in perceived well-being. Although this scale showed good internal consistency and was sensitive to change, further studies will be needed to validate this instrument.

## Data Availability

The data presented in this study are available in accordance with the ISS data access policy. Requests should be directed to Silvia Ciardullo (silvia.ciardullo@iss.it), National Centre for Disease Prevention and Health Promotion, Italian National Institute of Health, Rome, Italy.

## Abbreviations

COVID-19: Coronavirus disease 2019
SARS‑CoV‑2: Severe acute respiratory syndrome coronavirus 2
ISS: Istituto Superiore di Sanità
WHO: World Health Organization
PTSD: Post Traumatic Stress Disorder
COSI: Childhood Obesity Surveillance Initiative
LHU: Local Health Unit
WBS: Well-Being Score
SD: Standard Deviations
IQR: Interquartile range
OR: Odds Ratios
CI: Confidence Intervals
